# La pancréatite gravido-puerpérale: à propos de 6 cas

**DOI:** 10.11604/pamj.2015.20.185.4950

**Published:** 2015-02-27

**Authors:** Sarah Amourak, Sofia Jayi, Fatimazahra Fdili Alaoui, Hakima Bouguern, Hikmat Chaara, Moulay Abdelilah Melhouf

**Affiliations:** 1 Service de Gynécologie-obstétrique 2, CHU Hassan II, Fes, Université Sidi Mohammed Benabdellah

**Keywords:** Pancréatite, grossesse, vésicule lithiasique, lipasémie, cholécystéctomie, Pancreatitis, pregnancy, cholelithiasis, lipasemia, cholecystectomy

## Abstract

L'association pancréatite aigue et grossesse est rare mais non exceptionnelle, le diagnostic de la pancréatite au cours de la grossesse est difficile, vu la non spécificité de la symptomatologie et vu que très souvent les douleurs et les vomissements peuvent être rattachés à la grossesse elle-même pouvant ainsi être responsable du retard diagnostic L’échographie abdominale, qui est certes anodine pour le fœtus, ne permet pas de poser le diagnostic, par contre la TDM a une sensibilité, et une spécificité voisine de 100%, mais elle est irradiante elle n'est utilisable qu'au delà de 36SA et en post partum, et par conséquent l'IRM abdominale trouve tout son intérêt au 1er et au 2^ème^ trimestre. Cependant la biologie reste plus spécifique et c'est essentiellement l’élévation de la lipasémie à partir de 3fois la normale, qui peut redresser le diagnostic. Le traitement consiste au 1^er^ trimestre a une abstention thérapeutique et cholécystectomie au 2^ème^trimestre, au 2^ème^ trimestre: Cholécystectomie par cœlioscopie, au 3^ème^ trimestre: Sphinctérotomie, et en post partum, la cholécystectomie s'impose. A travers nos 6 cas et une revue de la littérature nous soulignons les caractéristiques diagnostiques, thérapeutiques et pronostiques de cette affection et nous invitons les praticiens à penser à la pancréatite chez toute femme enceinte avec douleur abdominales ce qui permettrait d’éviter les retards du diagnostic et d'améliorer le pronostic materno fœtal.

## Introduction

L'association pancréatite aigue et grossesse est rare mais non anecdotique avec une prévalence de 1 sur 3000 grossesses, vu le contexte gravidique cette entité peut poser un grand problème diagnostic mais aussi thérapeutique d'autant plus que le risque de récidive est important au cours de la même grossesse. A travers nos cas et une revue de la littérature nous soulignons les caractéristiques diagnostiques, thérapeutiques et pronostiques de cette affection.

## Méthodes

C'est une étude rétrospective analytique portant sur six observations de pancréatite au cours de la grossesse colligées sur une période de 3 ans (2010-2012) au service de gynécologie obstétrique II CHU Hassan II Fès.

## Résultats

Concernant les 6 cas colligés dans notre service, l’âge moyen des patientes est de 25ans, (23ans – 30ans). Une seule patiente uniquement a été connue porteuse d'une vésicule biliaire multi lithiasique, la pancréatite aigue est survenue chez une patiente au cours du 1er trimestre, chez 2 au cours du 2^ème^ trimestre et chez 3 patientes au cours du 3^ème^ trimestre. Les douleurs épigastriques et les vomissements représentent la symptomatologie commune chez toutes nos patientes.

### 1^ère^ observation

23 ans, G3P2, suivie pour VB multi lithiasique sous traitement symptomatique, enceinte à 18SA, qui a présenté des douleurs épigastriques intenses + vomissements, l'examen trouve une légère sensibilité épigastrique, lipasémie 10 fois la normale, écho ABD: aspect en faveur d'une pancréatite aigue. Elle a bénéficié d'une sphinctérotomie avec extraction d'un calcul de 7mm. L’écho ABD de contrôle a montré la persistance d'un calcul de 1cm au niveau du bas cholédoque, la patiente a été staffée avec les gastrologues pour une éventuelle reprise mais vu qu'elle s'améliorait sur le plan clinique et biologique la décision était une abstention thérapeutique au cours de la grossesse. Cependant elle a été perdue de vue

### 2^ème^ observation

30 ans, G3P2, sans antécédents pathologiques notables, enceinte se dit à terme, qui a présenté 2jours avant son admission des douleurs épigastriques + vomissement. L'examen trouve une patiente apyrétique, avec une légère sensibilité épigastrique, elle était en dehors du travail. La lipasémie était augmentée à 6 fois la normale, l’ échographie abdominale a objectivé une vésicule biliaire multi lithiasique à paroi fine. 12h après son admission elle est entrée spontanément en travail, elle a accouché par voie basse d'un Nné de sexe feminin, PN: 3100g, APGAR: 10/10. Le post partum était sans particularité, elle a été hospitalisé en réanimation, mise sous ration de base et arrêt de l'alimentation avec bonne évolution. Elle a bénéficié d'une TDM abdominale en post partum montrant une pancréatite stade C+ vésicule biliaire multi lithiasique, puis elle a été cholécystectomisée 2mois après.

### 3^ème^ observation

24 ans, G3P2, sans antécédents pathologiques notables, enceinte de 8mois, qui présente des douleurs épigastriques + vomissements. L'examen trouve une patiente stable sur le plan hémodynamique, apyrétique, et en dehors du travail. Lipasémie 10 fois la normale, l’échographie abdominale a montré une vésicule biliaire multi lithiasique à paroi fine, une bili IRM a montré un empierrement cholédocien, elle a été hospitalisé en réanimation avec ration de base et arrêt de l'alimentation, elle est entré spontanément en travail après 4j, avec accouchement prématuré par voie basse d'un nné de sexe masculin, APGAR 10/10, PN: 2400g, vu par les néonat et délivré à la famille. A j + 5 du post partum, elle a bénéficié d'une TDM abdominale montrant une pancréatite stade C. Elle a été programmée pour cholécystectomie réalisée à 2 mois du post partum.

### 4^ème^ observation

29 ans, G4P3, enceinte se dit à 2mois, admise pour des douleurs épigastriques avec des vomissements, l'examen clinique trouve une sensibilité épigastrique avec un utérus augmenté de taille faisant 8semaines d'aménorrhée. l’échographie abdominale était normale, et nous avons posé le diagnostic sur l’élévation de la lipasémie qui a été 31 fois la normale, une autre échographie abdominale refaite à 17SA est revenue normale. Elle a bénéficié d'un arrêt de l'alimentation, mise sous ration de base avec bonne évolution. Elle a été suivi jusqu’à 32semaines d'aménorrhée sans récidive puis elle a été perdu de vue.

### 5^ème^ observation

25 ans, primigeste, enceinte à 37semaines d'aménorrhée, qui a présenté 2jours avant son admission des vomissements+ douleur épigastrique. L'examen était son particularité, le bilan biologique a montré une lipasémie 10 fois la normale et l’échographie abdominale a objectivé une vésicule biliaire multi lithiasique, la TDM abdominale a stadifié la pancréatite en stade C. la patiente a bien évolué sous traitement symptomatique. Elle a bénéficié d'une cholécystectomie 2mois du post partum, après avoir accouché par voie basse un nouveau né de sexe féminin, PN:3kg100, APGAR:10/10.

### 6^ème^ observation

24 ans, primigeste, admise pour douleur épigastrique sur grossesse de 20semaine d'aménorrhée, l'examen trouve une patiente apyrétique, stable sur le plan hémodynamique, des BCF positifs et réguliers, contraction utérines négatives Le bilan biologique a montré une lipasémie a 15 fois la normale, l’échographie abdominale a objectivé une vésicule biliaire multi lithiasique: une IRM abdominale a stadifié la pancréatite en stade C et la patiente a bénéficié d'une CPRE avec une bonne évolution.

## Discussion

L'incidence de cette entité serait de 1/1000 à 1/11000, cette grande marge s'explique par la différence de critères diagnostiques tantôt cliniques, n'intéressant que les formes graves, tantôt biologiques, englobant même les formes bénignes [1, 2]. La pancréatite est rare au premier et deuxième trimestre (12%), alors qu'elle est fréquente au troisième trimestre (50%) et en postpartum (37%) (10). Il semble exister une relation de cause à effet entre pancréatite aiguë et état gravidique. Plusieurs théories sont évoquées: lithiasique, mécanique, par compression des canaux pancréatiques par l'utérus gravide, sécrétoire, métabolique [3, 4] (hyper triglycéridémie), endocrinienne (hyperparathyroïdie), vasomotrice (préeclampsie, hématome rétro placentaire) [3, 5].

Au cours de la grossesse surviennent des modifications physiologiques biliaires: la saturation en cholestérol, le volume vésiculaire, ainsi que la durée de la vidange vésiculaire sont augmentés au cours de la grossesse car ils sont directement influencé par l'augmentation du taux de la progestérone, L'ensemble de ces facteurs favorise la survenue de lithiase biliaire qui est de loin l’étiologie la plus fréquente des pancréatites aiguës gravidique comme c'est le cas chez nous [3].

### Diagnostic

Le diagnostic de la pancréatite au cours de la grossesse est difficile, vu la non spécificité de la symptomatologie et vu que très souvent les douleurs et les vomissements peuvent être rattachés à la grossesse elle-même pouvant ainsi être responsable du retard diagnostic. De plus L'examen clinique se trouve gêné par l'utérus gravide surtout au 2^ème^ et au 3^ème^trimestre, et la limitation des examens complémentaires non irradiant. Cependant la biologie reste plus spécifique et c'est essentiellement l’élévation de la lipasémie à partir de 3 fois la normale qui a une sensibilité et une spécificité voisine de 100% [6]. Le reste du bilan biologique (urée glycémie, calcémie…) a un intérêt surtout pronostique [7]. L’échographie abdominale, qui est certes anodine pour le fœtus, doit rechercher un élargissement du pancréas, des coulées de nécrose extra pancréatiques et une éventuelle lithiase biliaire. Néanmoins la TDM a une sensibilité, et une spécificité voisine de 100%, non seulement elle permet de poser le diagnostic de la pancréatite mais elle permet aussi de la stadifier, mais elle est irradiante elle n'est utilisable qu'au delà de 36SA et en post partum, et par conséquent l'IRM abdominale quand elle est disponible trouve tout son intérêt au 1er et au 2ème trimestre.

### Traitement

Le traitement médical est identique à la pancréatite en dehors de la grossesse: la mise à jeun, l'alimentation parentérale et le traitement de la douleur. Par contre le traitement spécifique de la pancréatite secondaire à une lithiase biliaire présente un réel problème de prise en charge, car d'une part il faut considéré le taux élevé des récidives au cours de la grossesse estimé à 70% versus 20 à 30% dans la population générale, et d'autre part la prise en charge doit considérer à la fois le risque maternel qui dépend du type anatomique de la pancréatite et de l’âge gestationnel de survenue, et le risque fœtal qui dépend de la gravité du tableau clinique, ce risque est représenté par l'avortement, la souffrance fœtale aigue et la mort fœtale in utéro qui est estimée à 15-25% [7]. L'intérêt de la cholécystectomie a été démontré par l'absence de complication maternelle ou foetale périopératoire. Dans une étude portant sur 24 cholécystectomies, toutes les cholécystectomies avaient été faites par laparotomie, 5 au premier trimestre, 11 au second, et 8 en post-partum. A l'opposé, les 61 autres patientes (85% de l'effectif) avaient été traitées médicalement, sans mortalité maternelle ou foetale. Cependant, une nouvelle poussée de PAB est survenue dans 69% des cas. Ces récidives ont nécessité cinq interventions chirurgicales en urgence [8].

Schematiquement le traitement doit être adapté au cas par cas en fonction de l’âge gestationnel: *1*^*er*^ *trimestre*: abstention thérapeutique et cholécystectomie au 2^ème^ trimestre; *2*^*ème*^ *trimestre*: Cholécystectomie par cœlioscopie car le risque de fausse-couche est diminué de moitié, passant de 12% (au premier trimestre) à 5,6% [9]; de même, le taux d'accouchement prématuré y est nul, alors qu'il est évalué à 40% lorsque l'intervention a lieu au troisième trimestre; *3*^*ème*^ *trimestre*: Sphinctérotomie; *En post partum*, la cholécystectomie s'impose.

### Pronostic

En 1973, le pronostic de la pancréatite aigue était encore très sévère, avec une mortalité foeto-maternelle évaluée à 37% [10]. Actuellement et grâce à la diminution des délais diagnostic et grâce aux progrès de la réanimation néonatale, la mortalité est actuellement nettement plus faible.

## Conclusion

La pancréatite aiguë est un événement certes rare chez la femme enceinte, mais qui peut engager le pronostic aussi bien maternel que fœtal. C'est la raison pour laquelle nous invitons les praticiens à penser à la pancréatite chez toute femme enceinte avec douleur abdominales ce qui permettrait d’éviter les retards du diagnostic et d'améliorer le pronostic materno fœtal.

## References

[1] Ramin K, Ramion S, Richey S, Cunningham R (1995). Acute pancreatitis in pregnancy. Am J Obstet Gynecol..

[2] Barrat J, Marpeau L, Pigue A (1986). Affection de l'appareil digestif. EMC Obstétrique.

[3] Medarhri J, Lekehal B, El Ounnani M, Ikken A, Echerrab M, Amraoui M, Errougani A, Benchekroun A, Chkoff R, Balafrej S (1997). Pancreatite et grossesse. Médecine du Maghreb.

[4] De Chalain TM, Michell WL, Berger GM (1988). Hyperlipidemia, pregnancy and pancreatitis. Surg Gynecol Obstet.

[5] Boyl JM, Macleod Me (1978). Pancreatic cancer presenting as pancreatitis of pregnancy. Am J Gastroenterol.

[6] Bahloul M, Ayedi M, Dammak H, Trabelsi K, Bouaziz M (2004). Pancréatite aigue nécrotico-hémorragique secondaire à une hypertension artérielle gravidique. Ann Fr Anesth Reanim..

[7] Jayi S, Bouguern H, Chaara H, Banani A, Melhouf MA (2008). A propos d'un syndrome pseudo occlusif au décours d'une césarienne. Espérance médicale.

[8] Pitchumoni CS, Yegneswaran B (2009). Acute pancreatitis in pregnancy. World J Gastroenterol..

[9] Jamidar PA, Beck GJ, Hoffman BJ (1995). Endoscopic retrograde cholangiopancreatography in pregnancy. Am J Gastroenterol..

[10] Wilkinson EJ (1973). Acute pancreatitis in pregnancy: a review of 98 cases and report of 8 new cases. Obstet Gynecol Surv..

